# Resistance band training with functional electrical stimulation improves force control capabilities in older adults: a preliminary study

**DOI:** 10.17179/excli2023-6777

**Published:** 2024-01-26

**Authors:** Joon Ho Lee, Hanall Lee, HyunJoon Kim, Rye-Kyeong Kim, Tae Lee Lee, Do-Kyung Ko, Hajun Lee, Nyeonju Kang

**Affiliations:** 1 Department of Human Movement Science, Incheon National University, Incheon, South Korea; 2 Division of Sport Science, Sport Science Institute, & Health Promotion Center, Incheon National University, Incheon, South Korea; 3 Neuromechanical Rehabilitation Research Laboratory, Division of Sport Science, Incheon National University, Incheon, South Korea

**Keywords:** strength training, resistance band, functional electrical stimulation, force control, older adults

## Abstract

Resistance band training (RBT) with functional electrical stimulation (FES) may be an effective exercise regimen for improving age-related motor impairments. This preliminary study investigated the potential effects of bimanual RBT with FES on upper limb motor functions in older adults. This study randomly assigned 22 elderly people to the bimanual RBT with FES (Bi-RBT+FES) group and the RBT without FES (Bi-RBT) group. All participants performed isometric hand-grip force control tasks in unimanual (dominant and non-dominant) and bimanual conditions before and after four weeks of exercise for each group. We quantified the mean force, force accuracy, force variability, and force regularity at two targeted force levels (i.e., 10 % and 40 % of maximum voluntary contraction; MVC) to estimate changes in force control capabilities. The results revealed that the Bi-RBT+FES group demonstrated a greater force accuracy in the dominant hand at 10 % of MVC after training. Non-dominant hands in the Bi-RBT+FES group increased force accuracy at 40 % of MVC and reduced force variability collapsed across two targeted force levels. Both groups showed a decrease in force regularity after training. These preliminary results indicate that Bi-RBT+FES may be a viable option to facilitate functional recovery of the upper limbs in older adults.

## Introduction

Aging facilitates progressive musculoskeletal and neurophysiological system changes (Roberts et al., 2016[55]; Tieland et al., 2018[65]). For example, decreased muscle fiber number and size appeared with greater synaptic noise and motor unit discharge rate variability for older adults (Hunter et al., 2016[21]). These age-related degenerations also cause abnormal patterns in muscle contraction that interfere with movement executions (Hunter et al., 2016[21]; Pethick et al., 2022[52]). In particular, upper extremity functional impairments in elderly people interfered with independently performing activities of daily living such as bathing, dressing, handling, and manipulating objects (Maes et al., 2017[42]). Thus, identifying optimal exercise protocols is necessary to effectively prevent age-related neuromuscular dysfunctions in the upper extremities.

Previous studies have frequently reported progressive muscle weakness, such as lower muscle strength and power compromising upper limb motor functions, in older adults (Halaweh, 2020[17]; Martin et al., 2015[45]; Vidt et al., 2012[70]). Strength exercise is generally recommended as a non-pharmacological intervention to improve neuromuscular functions (Chen et al., 2021[8]). The meta-analytic results indicated that strength training improved muscle mass, muscle strength, and functional performances in elderly people (Chen et al., 2021[8]; Grgic et al., 2020[15]; Peterson et al., 2010[51]). Importantly, specific strength exercise protocols for older adults should be carefully developed to minimize the risk of injury (Sousa et al., 2014[61]). Resistance band training (RBT) may be a feasible senior fitness option because older adults can perform strength exercises in multiple angles and planes effectively, thereby improving their muscle strength as well as neuromuscular functions (Colado and Triplett, 2008[11]). In fact, applying RBT to older adults improved muscle mass, strength, and joint flexibility of the upper limbs (Kim et al., 2022[31]; Liao et al., 2018[39]). Moreover, specific RBT protocols frequently involved bimanual movements because bimanual motor training could improve muscle strength by minimizing functional asymmetry between upper limbs via repetitive intermuscular coordinative actions (Liao et al., 2022[41]).

Functional electrical stimulation (FES) is a non-invasive electrical stimulation that may improve the efficiency of strength exercise by delivering low electrical pulses to a decentralized muscle via electrodes (Peckham and Knutson, 2005[50]). FES artificially induces the action potential and facilitates motor unit recruitments that potentially support voluntary muscle contractions (Barsi et al., 2008[3]; Joa et al., 2012[23]; van der Scheer et al., 2021[68]). Given that applying FES may be beneficial for improving the peripheral nervous system (e.g., muscle strength) as well as the central nervous system (e.g., greater sensorimotor cortical activations) (Hortobágyi and Maffiuletti, 2011[20]; Shin et al., 2022[58]), prior strength exercise protocols coupled with FES were frequently used for patients with neurological diseases (e.g., stroke) (Hara, 2008[18]; Kang et al., 2014[24]). For healthy older adults, only two studies have reported potential positive effects of strength exercise protocols coupled with neuromuscular electrical stimulation on motor functions of the lower limbs (Jang and Park, 2021[22]; Thapa et al., 2023[64]). These results indicated that FES, in addition to strength exercise, may facilitate upper extremity motor improvements; therefore, the combination of bimanual RBT and FES may be an effective option for the elderly population. Comparing treatment effect of bimanual RBT combined with FES relative to bimanual RBT only would increase our understanding of effective strength training protocols for functional recovery of aging population.

In this preliminary study, we investigated the effects of the combined protocols of bimanual RBT and FES on upper limb motor functions in healthy older adults compared with those who received bimanual RBT only. To estimate functional changes in the upper extremities, we focused on unimanual and bimanual isometric hand-grip force control capabilities (e.g., maximal force production and force control performances) frequently used for estimating neuromuscular functions in older adults (Keogh et al., 2010[28], 2019[30]). We hypothesized that older adults with bimanual RBT combined with FES would show greater motor improvements in the upper limbs than the elderly group with bimanual RBT only.

## Methods

### Participants

Twenty-two healthy older adults (16 females and 6 males; mean ± standard deviation of ages = 64.6 ± 3.3 years; range of mean age = 60.0-74.0 years) voluntarily participated in this study. To prevent potential effects of cognitive impairments on motor functions in older adults (Aggarwal et al., 2006[1]; Rudisch et al., 2020[56]; Schröter et al., 2003[57]), we confirmed that all participants had no musculoskeletal impairment, cognitive impairment, or neurological diseases. Further, we performed a priori power analysis using G*Power software (version 3.1.9.4), and confirmed that a minimum of 11 participants were necessary for a between-subjects design (power ≥ 0.96 and alpha = 0.05) (Faul et al., 2007[13]). Table 1(Tab. 1) shows the specific participant information. All participants read and signed an informed consent before the study participation. The Institutional Review Board of Incheon National University approved the study protocol (No. 7007971-201810-002A).

### Experimental procedures

We randomly assigned participants to the bimanual RBT with FES (Bi-RBT+FES) and the bimanual RBT without FES (Bi-RBT) groups. We instructed participants to avoid excessive physical activities and alcohol intake for 24 h and caffeine consumption and any medications (e.g., painkillers or sedatives) for 12 h before experimental testing and exercise session. Before starting the baseline test, all the training sessions were scheduled for each participant with specific description on the study protocol and potential adverse effects of FES (e.g., skin irritation, toleration, and acceptance issues) and RBT (e.g., slight muscle pain and fatigue) (Moll et al., 2017[47]). We provided four weeks of exercise programs (i.e., one session per week) for the Bi-RBT+FES and Bi-RBT groups, respectively.

### Resistance band training

**Figure 1:**
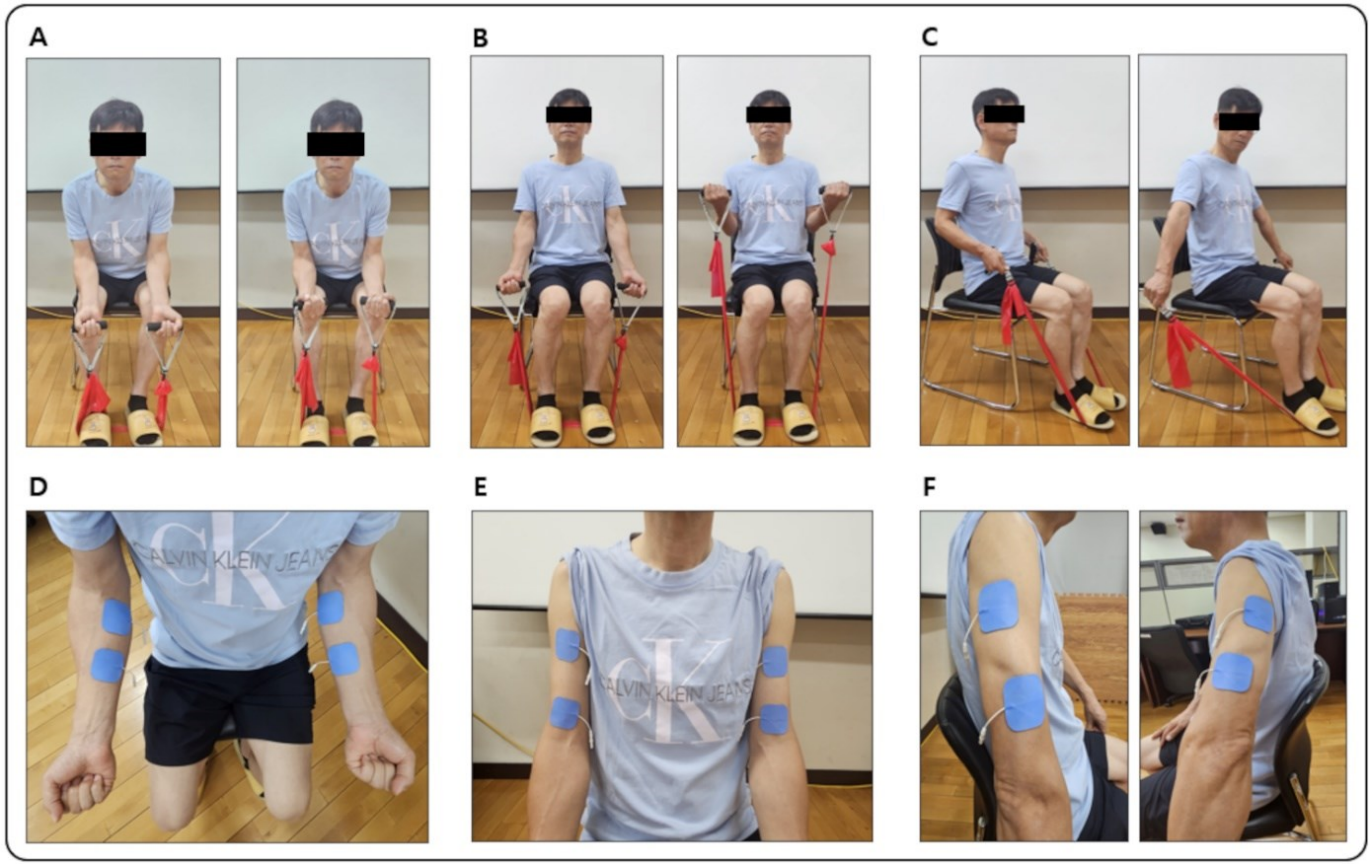
Resistance band training. Participants performing bimanual strength training using resistance band. (A) Wrist flexion. (B) Elbow flexion. (C) Elbow extension. (D) Electrodes attached to forearm muscles. (E) Electrodes attached to biceps brachii. (F) Electrodes attached to triceps brachii

For bimanual RBT, we used the elastic band (Theraband™, Naumcare, Sungnam, South Korea). A length of the elastic band was individualized based on a performer's ability to complete consecutive 10 repetitions for each bimanual movement condition. Each bimanual RBT session lasted approximately 60 min (i.e., warming up = 10 min, main exercise = 45 min, and cooling down = 5 min). Specific bimanual movements for RBT consisted of (a) wrist flexion, wherein the wrists are bimanually flexed while fixing both forearms on the armrest and pressing lower back and hips against a chair and maintaining a maximal wrist flexion for 5 s before returning slowly, (b) elbow flexion, wherein bimanually bicep curl is performed by raising the elastic band to a shoulder level with straightening back, fixing elbows, and holding the position for 5 s before returning to the starting position, and (c) elbow extension, wherein both elbows are fixed to each side of the trunk and bimanually extend both arms by holding grips of the elastic band until the peak resistance and maintaining the extended position for 5 s before returning to the initial position (Figure 1(Fig. 1)). For each participant, we individually administered all training protocols.

### Functional electrical stimulation

We used an FES device (Microstim2, SEJINMT©, Seoul, South Korea) to provide peripheral electrical stimulation in addition to bimanual RBT. The electrodes were attached to the forearm flexor, biceps brachii, and triceps brachii of both arms consistent with wrist flexion, elbow flexion, and elbow extension movements (Figure 1(Fig. 1)). We provided electrical stimulation when the participant initiated pulling the elastic band to perform bimanual movements. The FES protocol for the current study followed (a) pulse amplitude, which was set to an individualized level (i.e., a maximum level until feeling no uncomfortable pain during exercise), (b) pulse width, which was set to 200 *µ*s, (c) pulse frequency, which was set to 50 Hz, (d) timer, which was not implemented because of passively delivered stimulation, (e) stimulation period, which was set to 5 s, (f) ramp-up, wherein the pulse amplitude was gradually increased over 1 s at the initial of each active phase, (g) ramp-down, wherein the pulse amplitude was gradually decreased when participant returned to initial position, and (h) rest period, which is provided for 10 s between each active phase.

### Upper limb motor function assessments: isometric force control tasks

We applied isometric hand-grip force control paradigms before and after training to estimate upper limb motor function changes. We used the isometric hand-grip force measurement system (SEED TECH Co., Ltd., Bucheon, South Korea) for unimanual and bimanual force control tasks, and this device includes left and right handles (a diameter = 30 mm) that contain two force transducers on each side (Micro load Cell-CZL635-3135, range = 220 lbs, Phidgets Inc., Calgary, Canada). Participants seated 80 cm away from a 54.6 cm LED monitor (1920 × 1080 pixels; refresh rate = 60 Hz) and placed both arms on a table in comfortable positions (20°-45**°** of elbow flexion and 15°-20**°** of shoulder flexion).

**Figure 2:**
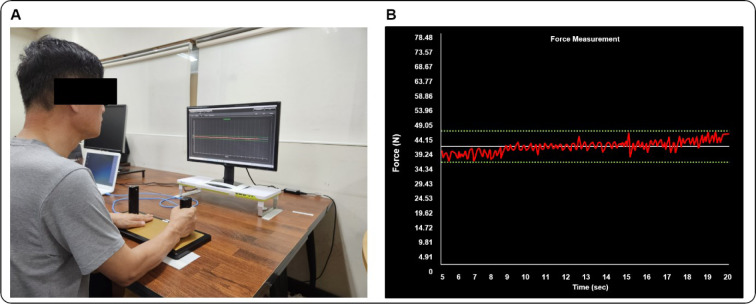
Experimental setup for upper limb force control tasks. (A) Participants used isometric hand-grip force measurement device to measure upper limb MVC and force control capabilities at individual targeted submaximal force levels (i.e., 10 % and 40 % of MVC). (B) Red line represents force signals produced by individual, white horizontal line indicates submaximal targeted force, and two parallel dotted green lines denote 10 % threshold from targeted force level.

Initially, participants completed two maximal voluntary contraction (MVC) trials (a trial duration = 5 s with 60 s of resting between trials) for unimanual (i.e., dominant and non-dominant hands) and bimanual conditions. We selected an average value of two peak forces (i.e., the maximum force output for each MVC trial) for each hand condition as the MVC of each participant. Submaximal muscle contractions (below 50 % of MVC) are typically required for conducting various daily activities (Marshall and Armstrong, 2004[44]; Rice et al., 2015[54]), and further prior studies revealed age-related force control deficits at these targeted force levels (Lee and Kang, 2023[38]; Strote et al., 2020[63]). Thus, we set two submaximal targeted force levels (i.e., 10 % and 40 % of MVC) (Hong et al., 2008[19]; Lee and Kang, 2023[38]; Slifkin et al., 2000[59]) for submaximal force control tasks so that participants were instructed to produce and maintain isometric forces (i.e., red line) to a targeted force level (i.e., white horizontal target line) for 20 s (Figure 2(Fig. 2)). Participants completed a total of four blocks of force control tasks for each condition (i.e., left and right hand; 10 % and 40 % force level) and each block consisted of three trials (total trial = 12) in the unimanual condition for each submaximal force control task (10 % and 40 % of MVC) in unilateral condition. Participants performed a total of four blocks for each submaximal targeted force level condition for the bimanual condition (i.e., 10 % and 40 % of MVC), and each block included three trials (total trials = 12). We provided 60 s of resting time to minimize muscle fatigue across trials.

We administered all experiment procedures using a custom Microsoft Visual C++ Program (Microsoft Corp., Redmond, WA, USA). Additionally, we sampled all data at the rate of 200 Hz with a 16-bit analog-to-digital converter (A/D; ADS1148 16-Bit 2kSPS and a minimum detectable force = 0.0192 N) and amplified them using INA122 with an excitation voltage of 5 V (Texas Instruments Inc., Dallas, TX, USA). We used the Matlab program (R2021a version, Math Works™ Inc., Natick, MA, USA) to conduct offline analyses.

### Data analysis

All of the raw force data were filtered through a bidirectional fourth-order Butterworth filter at a cut-off frequency of 30 Hz after acquiring the data (Math Works™ Inc., Natick, MA, USA). Additionally, we removed the first 3 s and last 3 s of each trial and focused on the middle 14 s of force data to minimize early adjustment and termination effects. The assessment of force control capabilities used the following outcome measures: (a) MVC and submaximal force production (mean force), (b) force accuracy: root-mean-square error (RMSE), (c) force variability: standard deviation (SD), and (d) force regularity: sample entropy (SampEn; see the Equation 1). For SampEn, the values close to zero denote more regular force production patterns, whereas greater SampEn values indicate lesser force regularity patterns.


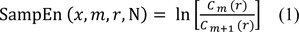


SampEn can be calculated using continuous variables (i.e., raw force data), where *m* represents specific patterns of lengths, *r *stands for a similarity criterion, and *C_m_* (*r*) demonstrates the prevalence of repetitive patterns of lengths *m *in time series but only* x* excludes match itself. We used 2 for *m *and* r* = 0.2 × SD of force production data based on previous studies (Vaillancourt et al., 2001[67]; Yentes et al., 2013[76]).

### Statistical analysis

Initially, we conducted the Shapiro-Wilk's test, and confirmed the normality of all dependent variables. For unimanual force control tasks, a three-way mixed analysis of variance (ANOVA) (group × time × hand; 2 × 2 × 2) on MVC and a four-way mixed ANOVA (group × time × hand × force level; 2 × 2 × 2 × 2) on submaximal mean force and force control variables (i.e., RMSE, SD, and SampEn) were performed. We conducted Bonferroni's pairwise comparisons for the post-hoc analyses. For bimanual force control tasks, a two-way mixed ANOVA on MVC (group × time; 2 × 2) and three-way mixed ANOVA (group × time × force level; 2 × 2 × 2) on submaximal mean force and force control variables were conducted. Bonferroni's pairwise comparisons were performed for the post-hoc analyses. We used the IBM Statistical Package for the Social Sciences version 25 (SPSS Inc., Chicago, IL, USA) for all statistical analysis procedures, and the alpha level was set at 0.05 for all outcome measures.

## Results

### MVC and submaximal force production

In unimanual condition, a three-way mixed ANOVA (group × time × hand; 2 × 2 × 2) on the MVC failed to identify any significant main and interaction effects. Additionally, a four-way mixed ANOVA (group × hand × time × force level; 2 × 2 × 2 × 2) on the submaximal mean force only showed the force level main effect (*F*_1, 20_ = 223.126; *P < *0.001; η^2^ = 0.918). Specifically, the values of submaximal mean force at 40 % of MVC (*M*±*SE* = 79.233±5.310) were significantly greater than 10 % of MVC (*M*±*SE* = 20.200±1.358). In bimanual condition, a three-way mixed ANOVA (group × time × force level) on the submaximal mean force showed a significant force level main effect (*F*_1, 18_ = 211.756; *P < *0.001; η^2^ = 0.922). The force level main effect results revealed that the submaximal mean force value at 40 % of MVC (*M*±*SE* = 159.829±10.950) was significantly greater than 10 % of MVC (*M*±*SE* = 40.806±2.773). These results indicate that applying bimanual RBT with FES did not alter the abilities to produce maximal and submaximal forces of unimanual and bimanual conditions in older adults (Supplementary information, Tables 1, 2, 6, and 7).

### Force control capabilities: force accuracy, force variability, and force regularity

**Figure 3:**
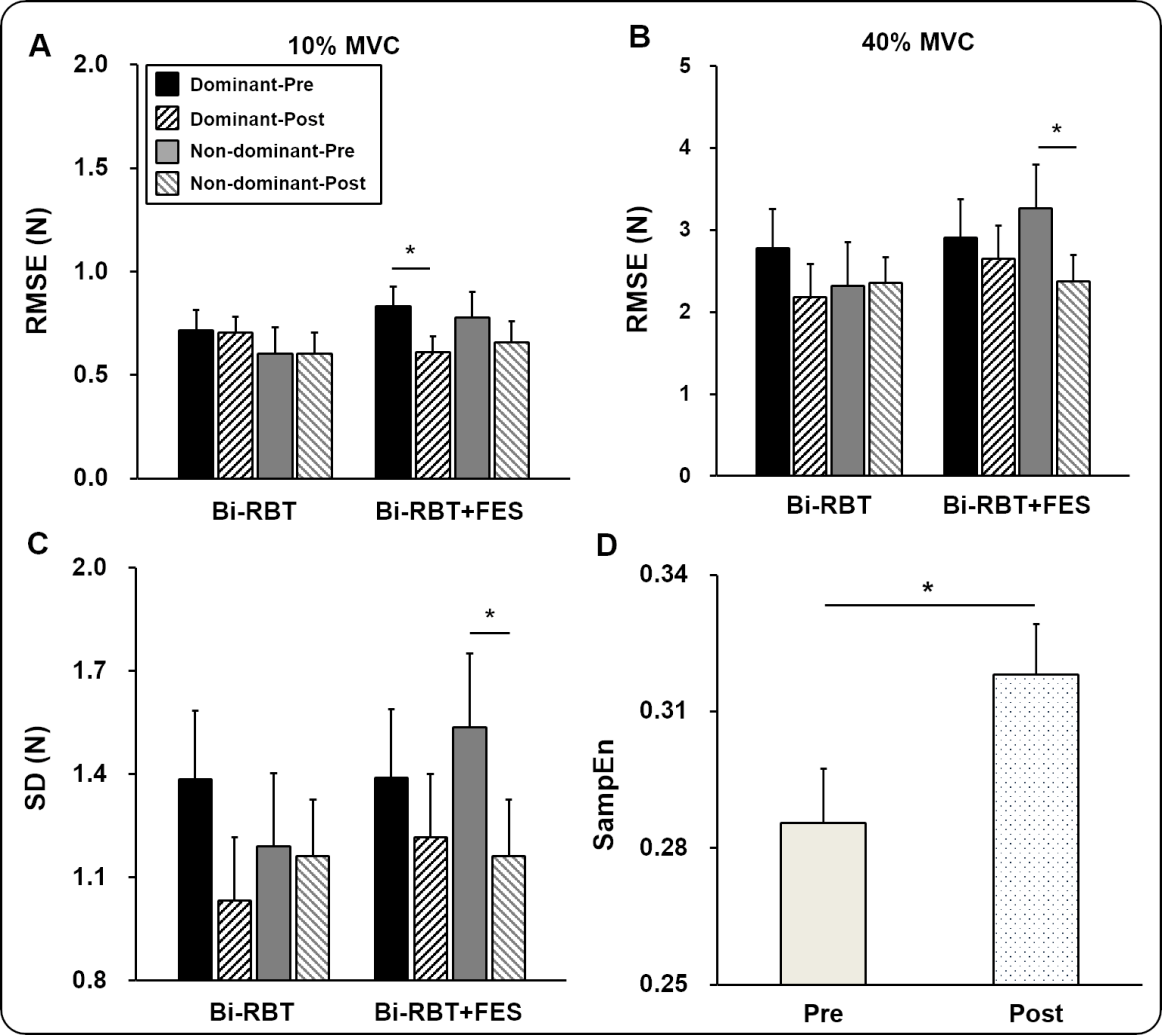
Force control capabilities for group, hand, time and force level conditions (*M*±*SE*). (A) Force accuracy (RMSE) revealing a significant group × hand × time × force level interaction effect. (B) RMSE revealing a significant group × hand × time × force level interaction effect. (C) Force variability (SD) showing a group × hand × time interaction effect. (D) Force regularity (SampEn) demonstrating a time main effect. *Asterisk* (*) indicates a significant difference between time condition (*P* < 0.005).

In unimanual condition, the analysis on the RMSE revealed a significant group × hand × time × force level interaction effect (*F*_1, 20_ = 4.824; *P = *0.040; η^2^ = 0.194). The post-hoc analyses indicated that the Bi-RBT+FES group demonstrated a significant reduction of RMSE in the dominant hand at 10 % of MVC (*P* = 0.037; Figure 3A(Fig. 3)) and in the non-dominant hand at 40 % of MVC (*P* = 0.039; Figure 3B(Fig. 3)), respectively. The SD analysis revealed a significant group × hand × time interaction effect (*F*_1, 20_ = 4.599; *P = *0.044; η^2^ = 0.187). The post-hoc analysis indicated that Bi-RBT+FES significantly decreased SD values in non-dominant hands collapsed across force level conditions (*P* = 0.020; Figure 3C(Fig. 3)). A four-way mixed ANOVA (group × hand × time × force level; 2 × 2 × 2 × 2) on the SampEn revealed two significant main effects: (a) time (*F*_1, 20_ = 11.956; *P = *0.002; η^2^ = 0.374) and (b) force level (*F*_1, 20_ = 304.150; *P *< 0.001; η^2^ = 0.938). Specifically, values of SampEn significantly increased after training collapsed across group, hand, and force level conditions (Figure 3D(Fig. 3)). However, the analysis failed to show any significant differences between groups (Supplementary information, Tables 3, 4, and 5).

In bimanual condition, three-way mixed ANOVAs (group × time × force level) showed significant force level main effects for (a) RMSE (*F*_1, 18_ = 62.295; *P *< 0.001; η^2^ = 0.776) and (b) SD (*F*_1, 18_ = 65.221; *P *< 0.001; η^2^ = 0.784). The analysis for SampEn revealed two main effects: (a) time (*F*_1, 18_ = 6.468; *P *= 0.020; η^2^ = 0.264) and (b) force level (*F*_1, 18_ = 224.463; *P *< 0.001; η^2^ = 0.926). Specifically, values of SampEn were significantly higher at posttest (*M*±*SE* = 0.279±0.012) in comparison to those at pretest (*M*±*SE* = 0.253±0.011). However, the analysis revealed no significant changes in force control capabilities between Bi-RBT+FES and Bi-RBT groups (Supplementary information, Tables 8, 9, and 10).

## Discussion

This preliminary study investigated the effects of Bi-RBT+FES on upper limb motor functions in healthy older adults compared to those who received Bi-RBT. All participants performed unimanual and bimanual isometric hand-grip force control tasks across maximal and submaximal levels at the pretest and posttest. The results revealed that the Bi-RBT+FES group showed greater force accuracy in the dominant hand at 10 % of MVC after training. Non-dominant hands in the Bi-RBT+FES group demonstrated better force accuracy at 40 % of MVC and lower force variability collapsed across two targeted force levels. Older adults for both groups produced less force regularity after training.

Force control capability improvements after bimanual strength training using elastic bands combined with FES expanded prior findings that bimanual RBT protocols facilitated functional upper extremity improvements in older adults (Kim et al., 2022[31], Liao et al., 2017[40], 2018[39]). Performing repetitive bimanual actions may be an effective exercise protocol for older adults to attenuate age-induced neuromuscular degeneration, which interferes with independent activities of daily living (Beurskens et al., 2015[4]; Noble et al., 2014[48]). Previous studies reported that bilateral movements increased cortical activations in each hemisphere (McCombe Waller et al., 2008[46]; Wu et al., 2021[75]), and functional connectivity patterns between the left and right sides of sensory-motor areas, premotor cortex, and primary motor cortex were facilitated after bilateral movement training (Grefkes et al., 2008[14]; Williams et al., 2010[72]). Moreover, applying FES during strength training may augment sensory neural signals that influence neural plasticity in the brain so that descending neural drives to both hands are presumably facilitated (Hortobágyi and Maffiuletti, 2011[20]; Shin et al., 2022[58]). These results indicated that Bi-RBT+FES may improve neuromuscular control capabilities in both hands with facilitated neural plasticity across the central and peripheral nervous systems (McCombe Waller et al., 2008[46]; Stoykov and Corcos, 2009[62]; Whitall et al., 2011[71]; Wu et al., 2021[75]).

A reduction of unimanual dominant hand force error at 10 % of MVC indicated that Bi-RBT+FES for older adults may be beneficial for improving force control capabilities in the dominant hand at a lower targeted level. These results supported previous findings that short-term (≤ four weeks) strength training with lower exercise intensity significantly improved force variability and manual dexterity in the dominant hand (Griffin et al., 2009[16]; Kornatz et al., 2005[33]; Laidlaw et al., 1999[36]; Marmon et al., 2011[43]). Many studies reported that older adults demonstrated more deficits in unimanual force control (e.g., greater force errors and variability) at lower targeted force levels (e.g., 2 %-20 % of MVCs) (Christou, 2011[9]; Christou and Enoka, 2011[10]; Keogh et al., 2006[27]; Laidlaw et al., 2000[35]; Lee et al., 2022[37]; Strote et al., 2020[63]). Thus, Bi-RBT+FES effectively improved fine motor control capabilities in older adults. Nevertheless, no significant changes in force control performances at 40 % of MVC may be related to insufficient training periods of Bi-RBT+FES improving muscle strength in the dominant hand.

For the non-dominant hand, Bi-RBT+FES effectively reduced force errors at 40 % of MVC and force variability collapsed across two targeted force levels. These results are consistent with previous findings that bimanual strength training decreased force variability in the non-dominant hand of older adults at a wide range of submaximal force levels (e.g., 10 %-65 % of MVCs) (Kavanagh et al., 2015[26]; Kobayashi et al., 2014[32]). Given that aging typically induces progressive neuromuscular degeneration in non-dominant hands because of increased motor dependence on their dominant hand (Krzysztofik et al., 2021[34]; Noguchi et al., 2009[49]), older adults may have more impairments in controlling force outputs in their non-dominant hand at various targeted force levels. Previous studies that used bimanual motor training with FES reported functional improvements in more affected hands (e.g., patients with stroke), and they indicated that these improvements may be associated with more comparable neural activation patterns between hemispheres after performing repetitive symmetrical movements between hands (Whitall et al., 2011[71]; Wu et al., 2010[74]). Similarly, the dominant hemisphere may positively influence the non-dominant hemisphere during Bi-RBT+FES, thereby contributing to improvements in overall non-dominant hand force control capabilities (Carson, 2005[6]; Cauraugh et al., 2010[7]; Williams et al., 2010[72]).

Despite no significant different changes in force regularity between the two groups, we confirmed that both groups showed a decrease in unimanual and bimanual force regularity after bimanual motor training. These results expanded a prior finding that unilateral upper limb strength training for older adults reduced force regularity, as indicated by greater SampEn values in dominant and non-dominant hand condition, respectively (Keogh et al., 2007[29]). Older adults typically revealed higher force regularity patterns during unimanual and bimanual isometric force control tasks compared to younger adults (Lee et al., 2022[37]; Lee and Kang, 2023[38]; Sosnoff and Newell, 2006[60]; Vaillancourt and Newell, 2003[66]). Aging may increase deficits in adjusting motor outputs in response to task and environmental constraints that presumably interfere with successful daily activities because more regular forces were related to stereotyped motor actions (Pethick et al., 2022[52], 2021[53]). Potentially, providing bimanual strength training for older adults may advance their force control adaptability.

Contrary to our hypothesis, we failed to identify positive effects of Bi-RBT+FES on bimanual force control capabilities in older adults. These patterns may be related to characteristics of bimanual RBT used for this study (e.g., both arms parallelly moved for resistance training). Successful bimanual force control normally requires coordinating interlimb motor actions in a synergistic way to achieve a task goal. Given that age-related neuromuscular changes impaired bimanual force control performances and coordination pattern (Kang et al., 2022[25]), previous studies suggested specific exercise programs involving more dynamic and complex interlimb movement executions (Arampatzis et al., 2011[2]; Dunsky, 2019[12]; Van Roie et al., 2020[69]; Wong et al., 2001[73]). Potentially, advancing Bi-RBT+FES intervention based on more cooperative interlimb actions during resistance training may improve bimanual force control capabilities in older adults.

Importantly, there are potential study limitations in this study. Although this preliminary study included 11 older adults per group based on the power analysis, investigating additional beneficial effects of Bi-RBT+FES with increased sample size is necessary to expand the current findings. Further, by including younger adult group, whether positive effects of Bi-RBT+FES specifically appear in aging population protocols should be determined in future studies. Next, considering a dose-response relationship between greater volume and period of exercise and muscle strength development in older adults (Borde et al., 2015[5]), how the Bi-RBT+FES intervention with higher volume and frequency of training sessions during longer periods affect the force control capabilities of older adults should be investigated.

In conclusion, this preliminary study showed that Bi-RBT+FES improved unimanual force control capabilities in older adults. After training, the Bi-RBT+FES group illustrated a significant reduction in force errors at 10 % of MVC on the dominant hand, and the non-dominant hand revealed a reduction in force errors at 10 % of MVC and force variability at 10 % and 40 % of MVCs. These results indicate that bimanual strength training using a resistance band with FES would be a viable option for motor recovery of older adults.

## Notes

Joon Ho Lee and Hanall Lee contributed equally as first author.

## Declaration

### Data availability statement

Correspondence and requests for materials should be addressed to NK.

### Ethics statement

The studies involving human participants were approved by the Incheon National University's Institutional Review Board (No. 7007971-201810-002A). Prior to the study, all participants read and signed a written informed consent.

### Authors' contributions

JHL, HL, HJK, RKK, TLL, DKK, and HJL contributed to data collection. JHL, HL, and NK were involved in statistical analyses, data interpretation, and manuscript drafts. NK conceived and designed the study. All authors read and approved the final manuscripts.

### Acknowledgments

The authors sincerely thank the study participants.

### Conflict of interest

The authors declare that they have no competing interests.

### Funding

This work was supported by a grant from the Ministry of Education of the Republic of Korea and the National Research Foundation of Korea (NRF- 2018R1C1B5084455 to NK).

## Supplementary Material

Supplementary information

## Figures and Tables

**Table 1:**
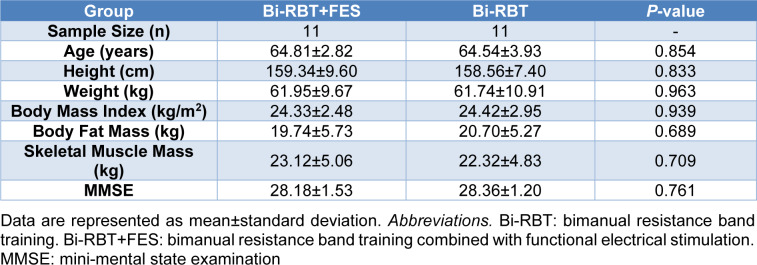
Participant characteristics
