# Supplementary material for: Resistance band training with functional electrical stimulation improves force control capabilities in older adults: a preliminary study
Source: EXCLI J. 2024 Jan 26;23:130–42. doi: 10.17179/excli2023-6777 (PMC10938250; doi:10.17179/excli2023-6777)
Supplement: Supplementary information [file EXCLI-23-130-s-001.pdf]

EXCLI Journal 2024;23:130-142 – ISSN 1611-2156
Received: November 07, 2023, accepted: January 05, 2024, published: January 26, 2024

Supplementary information to:

Original article:

RESISTANCE BAND TRAINING WITH FUNCTIONAL ELECTRICAL STIMULATION IMPROVES FORCE CONTROL CAPABILITIES IN OLDER ADULTS: A PRELIMINARY STUDY

Joon Ho Lee[a,b,c#], Hanall Lee[a,c#], HyunJoon Kim[a,c], Rye-Kyeong Kim[a,b,c], Tae Lee Lee[a,c], Do-Kyung Ko[a,c], Hajun Lee[a,c], Nyeonju Kang[a,b,c*]

[a] Department of Human Movement Science, Incheon National University, Incheon, South Korea
[b] Division of Sport Science, Sport Science Institute, & Health Promotion Center, Incheon National University, Incheon, South Korea
[c] Neuromechanical Rehabilitation Research Laboratory, Division of Sport Science, Incheon National University, Incheon, South Korea

[#] These authors contributed equally as first authors.

* **Corresponding author:** Nyeonju Kang, Ph. D., Neuromechanical Rehabilitation Research Laboratory, Division of Sport Science, Incheon National University, 119 Academy-ro, Yeonsu-gu, Incheon, South Korea. Phone: +82 32 835 8573, Fax: +82 32 835 0788, E-mail: nyunju@inu.ac.kr

https://dx.doi.org/10.17179/excli2023-6777

This is an Open Access article distributed under the terms of the Creative Commons Attribution License (http://creativecommons.org/licenses/by/4.0/).

EXCLI Journal 2024;23:130-142 – ISSN 1611-2156
Received: November 07, 2023, accepted: January 05, 2024, published: January 26, 2024

Supplementary Table 1: MVC descriptive statistics in unimanual task

Significance	*F*-value	*P*-value	η²
Group main effect	0.047	0.831	0.002
Time main effect	0.514	0.482	0.025
Time × Group interaction effect	0.012	0.915	0.001
Hand main effect	0.608	0.445	0.030
Hand × Group interaction effect	2.586	0.123	0.114
Time × Hand interaction effect	1.786	0.196	0.082
Time × Hand × Group interaction effect	2.961	0.101	0.129
**Conditions**	**Group**	**Mean±Standard Error**	
Dominant hand at pretest	Bi-RBT	213.55±18.24	
	Bi-RBT+FES	203.90±24.37	
Non-dominant hand at pretest	Bi-RBT	189.76±19.82	
	Bi-RBT+FES	209.51±26.50	
Dominant hand at posttest	Bi-RBT	195.18±14.48	
	Bi-RBT+FES	200.66±19.78	
Non-dominant hand at posttest	Bi-RBT	195.29±14.67	
	Bi-RBT+FES	203.26±23.08	

Abbreviations. Bi-RBT: bimanual resistance band training; Bi-RBT+FES: bimanual resistance band training combined with functional electrical stimulation; MVC: maximum voluntary contraction

EXCLI Journal 2024;23:130-142 – ISSN 1611-2156
Received: November 07, 2023, accepted: January 05, 2024, published: January 26, 2024

Supplementary Table 2: Mean Force descriptive statistics in unimanual task

Significance	*F*-value	*P*-value	η^2
Group main effect	0.033	0.858	0.002
Hand main effect	0.559	0.463	0.027
Hand × Group interaction effect	2.240	0.150	0.101
Time main effect	0.453	0.508	0.022
Time × Group interaction effect	0.033	0.858	0.002
**Force Level main effect**	**223.126**	**<0.001**	**0.918**
Force Level × Group interaction effect	0.032	0.861	0.002
Hand × Time interaction effect	1.881	0.185	0.086
Hand × Time × Group interaction effect	2.629	0.121	0.116
Hand × Force Level interaction effect	0.397	0.536	0.019
Hand × Force Level × Group interaction effect	2.348	0.141	0.105
Time × Force Level interaction effect	0.475	0.499	0.023
Time × Force Level × Group interaction effect	0.050	0.825	0.002
Hand × Time × Force Level interaction effect	1.760	0.200	0.081
Hand × Time × Force Level × Group interaction effect	2.831	0.108	0.124
**Conditions**	**Group**	**Mean±Standard Error**	
Dominant hand at 10 % of MVC at pretest	Bi-RBT	21.46±1.84	
	Bi-RBT+FES	20.50±2.40	
Dominant hand at 40 % of MVC at pretest	Bi-RBT	84.12±7.09	
	Bi-RBT+FES	79.88±9.37	
Dominant hand at 10 % of MVC at posttest	Bi-RBT	19.65±1.50	
	Bi-RBT+FES	20.13±1.98	
Dominant hand at 40 % of MVC at posttest	Bi-RBT	76.86±5.75	
	Bi-RBT+FES	78.89±7.79	
Non-dominant hand at 10 % of MVC at pretest	Bi-RBT	19.03±2.00	
	Bi-RBT+FES	20.82±2.62	
Non-dominant hand at 40 % of MVC at pretest	Bi-RBT	75.05±7.85	
	Bi-RBT+FES	81.94±10.16	
Non-dominant hand at 10 % of MVC at posttest	Bi-RBT	19.64±1.48	
	Bi-RBT+FES	20.36±2.32	
Non-dominant hand at 40 % of MVC at posttest	Bi-RBT	77.06±5.78	
	Bi-RBT+FES	80.05±9.08	

Abbreviations. Bi-RBT: bimanual resistance band training; Bi-RBT+FES: bimanual resistance band training combined with functional electrical stimulation; MVC: maximum voluntary contraction

EXCLI Journal 2024;23:130-142 – ISSN 1611-2156
Received: November 07, 2023, accepted: January 05, 2024, published: January 26, 2024

Supplementary Table 3: RMSE descriptive statistics in unimanual task

Significance	*F*-value	*P*-value	η^2
Group main effect	0.593	0.450	0.029
Hand main effect	0.106	0.748	0.005
Hand × Group interaction effect	0.308	0.585	0.015
Time main effect	2.507	0.129	0.111
Time × Group interaction effect	0.244	0.627	0.012
**Force Level main effect**	**82.113**	**<0.001**	**0.804**
Force Level × Group interaction effect	0.605	0.446	0.029
Hand × Time interaction effect	0.042	0.840	0.002
Hand × Time × Group interaction effect	5.612	0.028	0.219
Hand × Force Level interaction effect	0.140	0.712	0.007
Hand × Force Level × Group interaction effect	0.514	0.482	0.025
Time × Force Level interaction effect	1.161	0.294	0.055
Time × Force Level × Group interaction effect	0.180	0.676	0.009
Hand × Time × Force Level interaction effect	0.037	0.850	0.002
**Hand × Time × Force Level × Group interaction effect**	**4.824**	**0.040**	**0.194**
**Conditions**	**Group**	**Mean±Standard Error**	
Dominant hand at 10 % of MVC at pretest	Bi-RBT	0.71±0.09	
	Bi-RBT+FES	0.83±0.11	
Dominant hand at 40 % of MVC at pretest	Bi-RBT	2.78±0.36	
	Bi-RBT+FES	2.90±0.57	
Dominant hand at 10 % of MVC at posttest	Bi-RBT	0.60±0.08	
	Bi-RBT+FES	0.61±0.08	
Dominant hand at 40 % of MVC at posttest	Bi-RBT	2.19±0.27	
	Bi-RBT+FES	2.65±0.50	
Non-dominant hand at 10 % of MVC at pretest	Bi-RBT	0.71±0.07	
	Bi-RBT+FES	0.78±0.16	
Non-dominant hand at 40 % of MVC at pretest	Bi-RBT	2.32±0.28	
	Bi-RBT+FES	3.27±0.70	
Non-dominant hand at 10 % of MVC at posttest	Bi-RBT	0.60±0.08	
	Bi-RBT+FES	0.66±0.12	
Non-dominant hand at 40 % of MVC at posttest	Bi-RBT	2.35±0.27	
	Bi-RBT+FES	2.38±0.36	

Abbreviations. Bi-RBT: bimanual resistance band training; Bi-RBT+FES: bimanual resistance band training combined with functional electrical stimulation; MVC: maximum voluntary contraction; RMSE: root-mean-square error

EXCLI Journal 2024;23:130-142 – ISSN 1611-2156
Received: November 07, 2023, accepted: January 05, 2024, published: January 26, 2024

Supplementary Table 4: SD descriptive statistics in unimanual task

Significance	*F*-value	*P*-value	η^2
Group main effect	0.387	0.541	0.019
Hand main effect	0.007	0.932	<0.001
Hand × Group interaction effect	0.244	0.627	0.012
Time main effect	3.341	0.083	0.143
Time × Group interaction effect	0.107	0.747	0.005
**Force Level main effect**	**94.512**	**<0.001**	**0.825**
Force Level × Group interaction effect	0.237	0.631	0.012
Hand × Time interaction effect	0.256	0.619	0.013
**Hand × Time × Group interaction effect**	**4.599**	**0.044**	**0.187**
Hand × Force Level interaction effect	0.020	0.890	0.001
Hand × Force Level × Group interaction effect	0.365	0.553	0.018
Time × Force Level interaction effect	2.003	0.172	0.091
Time × Force Level × Group interaction effect	0.289	0.597	0.014
Hand × Time × Force Level interaction effect	<0.001	0.989	<0.001
Hand × Time × Force Level × Group interaction effect	3.319	0.083	0.142
**Conditions**	**Group**	**Mean±Standard Error**	
Dominant hand at 10 % of MVC at pretest	Bi-RBT	0.60±0.08	
	Bi-RBT+FES	0.67±0.08	
Dominant hand at 40 % of MVC at pretest	Bi-RBT	2.17±0.31	
	Bi-RBT+FES	2.11±0.36	
Dominant hand at 10 % of MVC at posttest	Bi-RBT	0.46±0.03	
	Bi-RBT+FES	0.52±0.06	
Dominant hand at 40 % of MVC at posttest	Bi-RBT	1.60±0.23	
	Bi-RBT+FES	1.92±0.40	
Non-dominant hand at 10 % of MVC at pretest	Bi-RBT	0.59±0.06	
	Bi-RBT+FES	0.65±0.14	
Non-dominant hand at 40 % of MVC at pretest	Bi-RBT	1.79±0.18	
	Bi-RBT+FES	2.42±0.45	
Non-dominant hand at 10 % of MVC at posttest	Bi-RBT	0.50±0.06	
	Bi-RBT+FES	0.57±0.09	
Non-dominant hand at 40 % of MVC at posttest	Bi-RBT	1.83±0.27	
	Bi-RBT+FES	1.75±0.28	

Abbreviations. Bi-RBT: bimanual resistance band training; Bi-RBT+FES: bimanual resistance band training combined with functional electrical stimulation; MVC: maximum voluntary contraction; SD: standard deviation

EXCLI Journal 2024;23:130-142 – ISSN 1611-2156
Received: November 07, 2023, accepted: January 05, 2024, published: January 26, 2024

Supplementary Table 5: SampEn descriptive statistics in unimanual task

Significance	*F*-value	*P*-value	η^2
Group main effect	0.100	0.755	0.005
Hand main effect	0.577	0.457	0.028
Hand × Group interaction effect	0.074	0.788	0.004
**Time main effect**	**11.956**	**0.002**	**0.374**
Time × Group interaction effect	0.009	0.927	<0.001
**Force Level main effect**	**304.150**	**<0.001**	**0.938**
Force Level × Group interaction effect	0.002	0.961	<0.001
Hand × Time interaction effect	1.142	0.298	0.054
Hand × Time × Group interaction effect	0.610	0.444	0.030
Hand × Force Level interaction effect	2.059	0.167	0.093
Hand × Force Level × Group interaction effect	0.968	0.337	0.046
Time × Force Level interaction effect	0.355	0.558	0.017
Time × Force Level × Group interaction effect	0.353	0.559	0.017
Hand × Time × Force Level interaction effect	0.034	0.856	0.002
Hand × Time × Force Level × Group interaction effect	0.195	0.664	0.010
**Conditions**	**Group**	**Mean±Standard Error**	
Dominant hand at 10 % of MVC at pretest	Bi-RBT	0.37±0.01	
	Bi-RBT+FES	0.35±0.02	
Dominant hand at 40 % of MVC at pretest	Bi-RBT	0.20±0.02	
	Bi-RBT+FES	0.21±0.02	
Dominant hand at 10 % of MVC at posttest	Bi-RBT	0.40±0.01	
	Bi-RBT+FES	0.39±0.01	
Dominant hand at 40 % of MVC at posttest	Bi-RBT	0.26±0.02	
	Bi-RBT+FES	0.24±0.01	
Non-dominant hand at 10 % of MVC at pretest	Bi-RBT	0.37±0.01	
	Bi-RBT+FES	0.37±0.02	
Non-dominant hand at 40 % of MVC at pretest	Bi-RBT	0.21±0.02	
	Bi-RBT+FES	0.19±0.02	
Non-dominant hand at 10 % of MVC at posttest	Bi-RBT	0.39±0.01	
	Bi-RBT+FES	0.39±0.02	
Non-dominant hand at 40 % of MVC at posttest	Bi-RBT	0.24±0.03	
	Bi-RBT+FES	0.23±0.02	

Abbreviations. Bi-RBT: bimanual resistance band training; Bi-RBT+FES: bimanual resistance band training combined with functional electrical stimulation; MVC: maximum voluntary contraction; SampEn: sample entropy

EXCLI Journal 2024;23:130-142 – ISSN 1611-2156
Received: November 07, 2023, accepted: January 05, 2024, published: January 26, 2024

Supplementary Table 6: MVC descriptive statistics in bimanual task

Significance	*F*-value	*P*-value	η^2
Group main effect	0.501	0.488	0.027
Time main effect	0.079	0.782	0.004
Time × Group interaction effect	0.018	0.894	0.001
**Conditions**	**Group**	**Mean±Standard Error**	
Bilateral MVC at pretest	Bi-RBT	388.78±40.38	
	Bi-RBT+FES	430.14±48.25	
Bilateral MVC at posttest	Bi-RBT	385.60±34.03	
	Bi-RBT+FES	421.05±43.29	

Abbreviations. Bi-RBT: bimanual resistance band training; Bi-RBT+FES: bimanual resistance band training combined with functional electrical stimulation; MVC: maximum voluntary contraction

EXCLI Journal 2024;23:130-142 – ISSN 1611-2156
Received: November 07, 2023, accepted: January 05, 2024, published: January 26, 2024

Supplementary Table 7: Mean Force descriptive statistics in bimanual task

Significance	*F*-value	*P*-value	η^2
Group main effect	0.398	0.536	0.022
Time main effect	0.034	0.856	0.002
Time × Group interaction effect	0.203	0.657	0.011
**Force Level main effect**	**211.756**	**<0.001**	**0.922**
Force Level × Group interaction effect	0.393	0.538	0.021
Time × Force Level interaction effect	0.058	0.812	0.003
Time × Force Level × Group interaction effect	0.230	0.637	0.013
**Conditions**	**Group**	**Mean±Standard Error**	
Bilateral hand at 10 % of MVC at pretest	Bi-RBT	39.24±4.03	
	Bi-RBT+FES	42.25±4.40	
Bilateral hand at 40 % of MVC at pretest	Bi-RBT	153.58±16.01	
	Bi-RBT+FES	165.04±17.29	
Bilateral hand at 10 % of MVC at posttest	Bi-RBT	38.85±3.43	
	Bi-RBT+FES	42.89±4.24	
Bilateral hand at 40 % of MVC at posttest	Bi-RBT	152.29±13.38	
	Bi-RBT+FES	168.40±16.91	

Abbreviations. Bi-RBT: bimanual resistance band training; Bi-RBT+FES: bimanual resistance band training combined with functional electrical stimulation; MVC: maximum voluntary contraction

EXCLI Journal 2024;23:130-142 – ISSN 1611-2156
Received: November 07, 2023, accepted: January 05, 2024, published: January 26, 2024

Supplementary Table 8: RMSE descriptive statistics in bimanual task

Significance	*F*-value	*P*-value	η^2
Group main effect	0.599	0.449	0.032
Time main effect	1.824	0.194	0.092
Time× Group interaction effect	0.270	0.610	0.015
**Force Level main effect**	**62.295**	**<0.001**	**0.776**
Force Level × Group interaction effect	0.560	0.464	0.030
Time × Force Level interaction effect	0.051	0.823	0.003
Time × Force Level × Group interaction effect	0.005	0.945	<0.001
**Conditions**	**Group**	**Mean±Standard Error**	
Bilateral hand at 10 % of MVC at pretest	Bi-RBT	1.11±0.12	
	Bi-RBT+FES	1.08±0.17	
Bilateral hand at 40 % of MVC at pretest	Bi-RBT	3.88±0.53	
	Bi-RBT+FES	4.41±0.66	
Bilateral hand at 10 % of MVC at posttest	Bi-RBT	0.83±0.12	
	Bi-RBT+FES	0.96±0.19	
Bilateral hand at 40 % of MVC at posttest	Bi-RBT	3.51±0.56	
	Bi-RBT+FES	4.24±0.69	

Abbreviations. Bi-RBT: bimanual resistance band training; Bi-RBT+FES: bimanual resistance band training combined with functional electrical stimulation; MVC: maximum voluntary contraction; RMSE: root-mean-square error

EXCLI Journal 2024;23:130-142 – ISSN 1611-2156
Received: November 07, 2023, accepted: January 05, 2024, published: January 26, 2024

Supplementary Table 9: SD descriptive statistics in bimanual task

Significance	*F*-value	*P*-value	η^2
Group main effect	0.144	0.709	0.008
Time main effect	2.175	0.158	0.108
Time× Group interaction effect	0.999	0.331	0.053
**Force Level main effect**	**65.221**	**<0.001**	**0.784**
Force Level × Group interaction effect	0.076	0.786	0.004
Time × Force Level interaction effect	0.553	0.467	0.030
Time × Force Level × Group interaction effect	0.522	0.479	0.028
**Conditions**	**Group**	**Mean±Standard Error**	
Bilateral hand at 10 % of MVC at pretest	Bi-RBT	0.79±0.11	
	Bi-RBT+FES	0.79±0.11	
Bilateral hand at 40 % of MVC at pretest	Bi-RBT	2.94±0.46	
	Bi-RBT+FES	2.91±0.40	
Bilateral hand at 10 % of MVC at posttest	Bi-RBT	0.64±0.07	
	Bi-RBT+FES	0.73±0.12	
Bilateral hand at 40 % of MVC at posttest	Bi-RBT	2.44±0.37	
	Bi-RBT+FES	2.85±0.47	

Abbreviations. Bi-RBT: bimanual resistance band training; Bi-RBT+FES: bimanual resistance band training combined with functional electrical stimulation; MVC: maximum voluntary contraction; SD: standard deviation

EXCLI Journal 2024;23:130-142 – ISSN 1611-2156
Received: November 07, 2023, accepted: January 05, 2024, published: January 26, 2024

Supplementary Table 10: SampEn descriptive statistics in bimanual task

**Significance**	***F*-value**	***P*-value**	**η^2**
Group main effect	0.575	0.458	0.031
**Time main effect**	**6.468**	**0.020**	**0.264**
Time× Group interaction effect	0.124	0.729	0.007
**Force Level main effect**	**224.463**	**<0.001**	**0.926**
Force Level × Group interaction effect	0.701	0.413	0.038
Time × Force Level interaction effect	0.620	0.441	0.033
Time × Force Level × Group interaction effect	0.984	0.334	0.052
**Conditions**	**Group**	**Mean±Standard Error**	
Bilateral hand at 10 % of MVC at pretest	Bi-RBT	0.34±0.02	
	Bi-RBT+FES	0.33±0.02	
Bilateral hand at 40 % of MVC at pretest	Bi-RBT	0.18±0.02	
	Bi-RBT+FES	0.16±0.01	
Bilateral hand at 10 % of MVC at posttest	Bi-RBT	0.37±0.02	
	Bi-RBT+FES	0.37±0.03	
Bilateral hand at 40 % of MVC at posttest	Bi-RBT	0.21±0.02	
	Bi-RBT+FES	0.17±0.01	

Abbreviations. Bi-RBT: bimanual resistance band training; Bi-RBT+FES: bimanual resistance band training combined with functional electrical stimulation; MVC: maximum voluntary contraction; SampEn: sample entropy
